# 4323 Inflammation partially mediates fatigue-like behavior in mice

**DOI:** 10.1017/cts.2020.71

**Published:** 2020-07-29

**Authors:** Sarah Alshawi, Brian Wolff, Leorey Saligan

**Affiliations:** 1 National Institutes of Health

## Abstract

OBJECTIVES/GOALS: Fatigue is a distressing side effect of cancer and its treatment. It is a subjective symptom that can include mental, physical, emotional, and motivational components. We sought to determine whether preventing inflammation affects fatigue-like behavior in a mouse model of radiation therapy. METHODS/STUDY POPULATION: C57BL/6 mice received three consecutive 8 gray doses of daily peripheral irradiation. We used voluntary wheel running activity to measure fatigue-like behavior before and after this period. Minocycline, an antibiotic with anti-inflammatory effects, was administered beginning a week before irradiation and continued throughout the experiment. We also tested mice lacking the toll-like receptor adaptor protein, MyD88. Cognitive abilities were tested using spontaneous alternation in a Y-maze. RESULTS/ANTICIPATED RESULTS: We found that minocycline reduces fatigue-like behavior exhibited after irradiation, but had no effect on pre-irradiation activity levels. Similarly, fatigue-like behavior after radiation was partially reversed by genetic loss of MyD88. Y-maze spontaneous alternation performance remained similar in all groups. DISCUSSION/SIGNIFICANCE OF IMPACT: Both pharmacological and genetic anti-inflammatory manipulations increased voluntary activity levels after irradiation. Our results suggest that inflammation is an important factor in the development of fatigue-like behavior. Modulators of inflammatory processes hold potential for alleviating fatigue.

